# “They are human beings, they are Swazi”: intersecting stigmas and the positive health, dignity and prevention needs of HIV-positive men who have sex with men in Swaziland

**DOI:** 10.7448/IAS.16.4.18749

**Published:** 2013-12-02

**Authors:** Caitlin E Kennedy, Stefan D Baral, Rebecca Fielding-Miller, Darrin Adams, Phumlile Dludlu, Bheki Sithole, Virginia A Fonner, Zandile Mnisi, Deanna Kerrigan

**Affiliations:** 1 Department of International Health, Johns Hopkins Bloomberg School of Public Health, Baltimore, MD, USA; 2 Department of Epidemiology, Johns Hopkins Bloomberg School of Public Health, Baltimore, MD, USA; 3 Department of Health Behavior, Emory University School of Public Health, Atlanta, GA, USA; 4 Futures Group, Washington, DC, USA; 5 Mbabane, Swaziland; 6 University of Stellenbosch, Stellenbosch, South Africa; 7 Ministry of Health and Social Welfare, Mbabane, Swaziland; 8 Department of Health, Behavior and Society, Johns Hopkins Bloomberg School of Public Health, Baltimore, MD, USA

**Keywords:** men who have sex with men, positive health dignity and prevention, people living with HIV, qualitative research, Swaziland

## Abstract

**Introduction:**

Despite the knowledge that men who have sex with men (MSM) are more likely to be infected with HIV across settings, there has been little investigation of the experiences of MSM who are living with HIV in sub-Saharan Africa. Using the framework of positive health, dignity and prevention, we explored the experiences and HIV prevention, care and treatment needs of MSM who are living with HIV in Swaziland.

**Methods:**

We conducted 40 in-depth interviews with 20 HIV-positive MSM, 16 interviews with key informants and three focus groups with MSM community members. Qualitative analysis was iterative and included debriefing sessions with a study staff, a stakeholders’ workshop and coding for key themes using Atlas.ti.

**Results:**

The predominant theme was the significant and multiple forms of stigma and discrimination faced by MSM living with HIV in this setting due to both their sexual identity and HIV status. Dual stigma led to selective disclosure or lack of disclosure of both identities, and consequently a lack of social support for care-seeking and medication adherence. Perceived and experienced stigma from healthcare settings, particularly around sexual identity, also led to delayed care-seeking, travel to more distant clinics and missed opportunities for appropriate services. Participants described experiences of violence and lack of police protection as well as mental health challenges. Key informants, however, reflected on their duty to provide non-discriminatory services to all Swazis regardless of personal beliefs.

**Conclusions:**

Intersectionality provides a framework for understanding the experiences of dual stigma and discrimination faced by MSM living with HIV in Swaziland and highlights how programmes and policies should consider the specific needs of this population when designing HIV prevention, care and treatment services. In Swaziland, the health sector should consider providing specialized training for healthcare providers, distributing condoms and lubricants and engaging MSM as peer outreach workers or expert clients. Interventions to reduce stigma, discrimination and violence against MSM and people living with HIV are also needed for both healthcare workers and the general population. Finally, research on experiences and needs of MSM living with HIV globally can help inform comprehensive HIV services for this population.

## Introduction

Globally, men who have sex with men (MSM) have substantially higher levels of HIV infection than men in the general population [1]. This is true even in the generalized HIV epidemics of sub-Saharan Africa, where MSM have more than three times the HIV prevalence of general population adult males on average [1]. Despite the knowledge that MSM are more likely to be infected with HIV across settings, there has been little investigation of the experiences of MSM who are living with HIV in sub-Saharan Africa.

Positive health, dignity and prevention is a framework used to highlight health and social justice issues for people living with HIV (PLHIV) [2,3]. The primary goals of positive health, dignity and prevention are “to improve the dignity, quality, and length of life of people living with HIV; which, if achieved will, in turn, have a beneficial impact on their partners, families, and communities, including reducing the likelihood of new infections” [2]. This framework builds upon earlier concepts of “positive prevention” and “prevention with positives,” which highlighted the importance of ensuring the health of PLHIV and engaging PLHIV in HIV-prevention efforts [4–6]. However, positive health, dignity and prevention situates living with HIV within a human rights framework and focuses on the importance of understanding and addressing structural constraints. It also considers the role of stigma and discrimination, which Parker and Aggleton [7] describe as social processes related to social inequality, power and oppression through which some groups are structurally excluded in society. Stigma has often been defined based on the classic work of Goffman as the social devaluation of a person based on a “significantly discrediting” attribute [8], while discrimination has been defined as behaviour resulting from prejudice [9]. Both stigma and discrimination are common in relation to both HIV and same-sex relationships.

In Swaziland, HIV prevalence in reproductive-age adults is among the highest in the world at 26.1% [10]. UNAIDS classifies Swaziland as a generalized HIV epidemic and, to date, the response to HIV in Swaziland has largely focused on the general population. Recently, the first surveillance of HIV prevalence and associated risk factors among MSM in Swaziland was conducted and showed a high burden of HIV among Swazi MSM, comparable to that of men in the general population [11]. However, same-sex behaviour is criminalized in Swaziland, and little attention has focused on the experiences of MSM who are living with HIV in this setting. Indeed, we identified just one peer-reviewed article focusing on HIV-positive MSM in sub-Saharan Africa. Cloete *et al*. [12] conducted a survey on HIV-related stigma and discrimination among a convenience sample of both HIV-positive MSM and men who have sex with women in Cape Town, South Africa. The survey found that internalized HIV-related stigma was high among all participants. Overall, MSM reported slightly greater social isolation and discrimination due to their HIV status, but these differences generally did not reach statistical significance.

In this study, we sought to explore the positive health, dignity and prevention needs of MSM who are living with HIV in Swaziland to inform HIV prevention, care and treatment services for this population. To our knowledge, this is one of the first qualitative studies to examine these issues among HIV-positive MSM in sub-Saharan Africa. As such, findings could inform the design and implementation of programmes for MSM living with HIV in Swaziland and similar settings.

## Methods

A qualitative approach was used to address the study aims. Methods included key informant interviews, in-depth interviews with HIV-positive MSM, and focus groups with MSM community members.

Key informants were selected if they had experience with MSM and lesbian, gay, bisexual and transgender (LGBT) populations or with HIV-related services in Swaziland. Sixteen key informants were interviewed, including HIV programme planners, policy makers, clinicians and LGBT community leaders. Interviews were semi-structured and employed a field guide to direct the conversation and stimulate probing. Participants were asked to describe the situation of MSM in their communities, their knowledge of existing services for MSM and PLHIV and their suggestions for how services could better meet the needs of MSM.

In-depth interviews were conducted with 20 MSM living with HIV interviewed twice each for a total of 40 interviews. Recruitment was conducted through a variety of settings and organizations, including HIV clinics; PLHIV networks; LGBT and MSM community organizations; and HIV prevention, care and treatment services. Participants were asked about the experiences of MSM generally in their communities; MSM social networks; personal and community experiences with HIV prevention, care and treatment services; experiences with stigma and discrimination; and suggestions for how services, interventions and messages could be better tailored for MSM.

Focus groups were conducted with MSM to gather a broader community perspective on the study topics; HIV status was not asked for reasons of confidentiality. Three focus groups were conducted with 26 MSM (4, 9 and 13 participants in each group). Topics covered were similar to interviews.

All interviews and focus groups were conducted in a private setting in either English or SiSwati and lasted approximately one to two hours. MSM were interviewed by a Swazi familiar with the local LGBT community who received training in qualitative research, while key informants were interviewed by an American masters-level research assistant with qualitative training living in Swaziland.

### Qualitative data analysis

Analysis of qualitative data was conducted through identification of recurrent patterns and themes following Crabtree and Miller's five steps in qualitative data analysis, or the “interpretive process” [13]. These steps are: (i) describing, (ii) organizing, (iii) connecting, (iv) corroborating and (v) representing. These steps form part of an iterative process which starts by re-examining the goals of the research and considering questions of reflexivity, then moves towards ways of highlighting, arranging and reducing texts to make connections through the identification of recurrent patterns and themes.

All interviews and focus groups were recorded, transcribed and translated into English. Debriefing notes immediately following each interview captured the interview context, theoretical issues, methodological issues and follow-up topics. Weekly meetings were held with all interviewers to discuss emerging themes and identify topics for further exploration to ensure an iterative process. After all data were collected, a full-day data analysis workshop was attended by representatives from LGBT groups, Ministry of Health (MOH) and National Emergency Response Council on HIV and AIDS (NERCHA) representatives, interviewers, clinicians and other stakeholders. This workshop devoted individual time to read de-identified transcripts to identify themes, then group time to categorize and discuss emerging themes and implications. Following the workshop, a codebook was developed by four study team members working together until agreement was reached. Codes were selected based on *a priori* topics of interest (research questions), themes identified during the data analysis workshop and emergent themes from transcripts. Codes were then applied using the computer software package Atlas.ti (version 5.2, Scientific Software Development GmbH, Eden Prairie, MN). The coded text was read to identify further themes or patterns and memos were created for key themes, which were developed into the findings presented here.

### Ethical considerations

All participants provided oral informed consent prior to participation, and referrals to clinical and counselling services were provided as needed. Study staff members were trained on sensitivity issues around HIV and MSM. A study advisory board, including representation from the LGBT community, implementing partners and government, reviewed the study protocol and interview guides and provided ongoing advice to the management and execution of the study. Ethical review and approval for this study was received from the Scientific and Ethics Committee of the Swaziland MOH and the Johns Hopkins Bloomberg School of Public Health in the United States.

## Results

### Dual stigma and disclosure of sexual identity and HIV status

The predominant theme across interviews was the significant and multiple forms of stigma and discrimination faced by MSM living with HIV in Swaziland. MSM reported experiencing stigma and discrimination related to both their HIV status as well as their sexual identity.

Same-sex behaviour is both criminalized and heavily stigmatized in Swaziland. MSM reported experiencing significant stigma, discrimination and rejection as a result of their sexual identity. One man, when asked if he had ever experienced stigma or discrimination as a result of being gay, responded, “A lot, several times, too many times.”

As a result of these experiences, and fear of similar stigma and rejection, many participants said they had not disclosed their sexual identity to anyone except other MSM. “That is my secret and I'm not planning to tell anyone in my family,” explained one. Participants worried about negative reactions, rejection and abuse if they disclosed. One man, when asked what would happen if he disclosed his sexuality to his friends or family, responded, “I would not even dare. It would be like being in a devil's den.” Others worried more about disappointing their loved ones by not conforming to social norms. One MSM asked,

Do you know this SiSwati saying that goes, ‘you have to have a heart for the other person’? … We always put the next person before [ourselves] … So we hardly want to disappoint the next person with being me, being myself and being comfortable with myself and insisting that I should be accepted, you know. We want to always conform [to] what society expects.

However, some participants had disclosed their sexual identity to family members or friends and had found acceptance, often after some initial difficulty.

Men also described stigma related to their HIV status. One participant described “the abuse we are subjected to” as “stigma, you see, that once you are HIV-positive, people think that you have AIDS. And also, that people have not accepted and they still do not know what HIV is.” Experiences or fear of HIV-related stigma prevented many MSM from disclosing their HIV status to family, friends and sexual partners. Lack of disclosure led to challenges with antiretroviral drug (ARV) adherence, hiding medications and a lack of social support for care-seeking and adherence to care and ARVs.

Participants selectively disclosed either their HIV status or their sexual identity to different individuals based on their anticipated reaction. For example, participants said they might disclose their HIV status to family members as they anticipated receiving some material or emotional support as a result, but they might not disclose their sexual identity to those same family members due to fear of rejection or a negative reaction.

### Violence and lack of police protection

Violence was also a common experience for MSM. MSM reported violence from a range of individuals. One man noted that some MSM “are killed for being gay, others are assaulted and others are chased away from home and disowned.” Due to the criminalized nature of same-sex behaviour in Swaziland, many MSM felt they had no recourse to bring incidents of discrimination or violence to the authorities. Furthermore, many had experienced a lack of police protection as a result of their sexuality. One participant described such an incident:

Participant (P): I was actually with a friend of mine in Manzini and we went to the butchery for a braai [barbecue], and when we got there, umm, there were these people who were, like, sitting outside at the car park. They were just rude and they started insulting us and we didn't try to defend ourselves, try to explain anything, and they went on, like, we are gay, we have to be beaten up, the gayness should be beaten out of us. We just ignored them and they attacked one of my friends we were with, they started beating him and he was bleeding.

Interviewer (I): Really.

P: Like for real, he bled to the point where we had to go to the hospital and we obviously went to lay a charge. And the police were kind of ‘occupied’, they didn't have the time to go and find these people that have beaten my friend.

I: Why did the police act in that way? Did you narrate to them what happened?

P: We sure did, but I think it's because we told them how the whole thing started – they called us names because they say we are gay. And I think also the police could tell that we are [gay], so they thought there was no case there.

### Stigma from healthcare settings

The stigma associated with being an MSM was the predominant barrier to accessing healthcare services for MSM living with HIV. Both perceived and experienced stigma in healthcare settings led to a lack of care-seeking behaviour. As one participant described it,

When they say ‘bring your partner’, and then you bring the same sex partner, they are like, ‘yah, this is why you are having this [HIV], this is why’, and they will be throwing words at you … so then you get embarrassed, sometimes you'll decide to leave without being treated, and where are you taking that sickness to?

Another participant, when asked how the needs of MSM differed from PLHIV in general, explained that the main difference was how forthright MSM could be about issues related to their sexuality:

I think they are different in the sense that for those who are straight they are open and they communicate easily about sex issues. As for us gays, it's difficult unless you have someone you can talk to and give you advice as to what you can do when you have some health issues. As for people in general, with them it's easy for them to go to hospital, but with us it's difficult. You can't say it's painful in your anus – what will you say the cause for that is?

This participant continued by noting that this influenced care-seeking behaviour, as he would delay care-seeking or self-medicate to avoid disclosure:

I: What happens, so you end up not going there [to the hospital]?

P: I just stay at home and you find that this thing becomes complicated. When this thing becomes complicated, you find that maybe you go to the pharmacy and they tell you that this thing is at an advanced stage.

Other men said they travelled long distances to seek HIV care at clinics where they either were not known personally or where they did not experience stigma and discrimination.

P: Even at the hospital, they interviewed me, then there were changes and I could tell that they wanted me to reveal what type of person I am. Since then I stopped fetching my drugs there. I now go to another clinic which is far away from home. I drive all the way to fetch my tablets instead of taking them locally.

I: Really, why is it so?

P: Because I thought there is problem at the local clinic since I am gay. So I decided to change … They treat us like small devils, as if we are the one who are spreading the HIV virus.

However in a few cases, MSM did disclose their sexual identity to healthcare providers and reported positive and supportive reactions, particularly from non-governmental HIV testing and counselling sites.

Fear of stigma also shaped the type and nature of counselling that MSM received in healthcare settings, particularly regarding offering services to sexual partners. MSM, as well as key informants, noted that in clinical services such as HIV testing and treatment, providers’ questions about HIV prevention generally assume heterosexuality. Providers would ask MSM to bring their wives into the clinic to be tested for HIV. Due to fear of stigma, MSM would often simply state that they did not have a wife, but would not mention their male sexual partners.

Finally, participants reported mistreatment by staff and lack of confidentiality at clinics due to being HIV-positive. These negative experiences were particularly experienced when picking up ARVs, leading one MSM to say that “the ARVs end up being an inconvenience [rather] than helping you.” Some men felt that PLHIV in general were treated poorly by healthcare workers. “You really feel that you are different from other people,” explained one. However, others felt that at least some healthcare workers provided high-quality care to PLHIV, and that MSM were not necessarily treated any differently from other PLHIV.

### Mental health challenges

Many MSM said that living with a stigmatized sexual identity and a challenging, stigmatized disease led to feelings of depression as well as self-stigma or shame. “To be like this to me seems like I was created for nothing on earth,” said one, “because there is nobody who is happy about me at home and at school.”

The initial receipt of an HIV-positive diagnosis was emotionally devastating for many participants. Participants described feelings of depression and anger. They also said that others had even more difficult coping. “Some of them they commit suicide because they can't accept their status,” said one MSM, “because no one can accept them as they are gay and positive.” Some participants said feelings of self-stigma led them to drink alcohol as a coping mechanism.

[After testing HIV-positive], I was very much hurt so much that I decided to devote myself to drinking alcohol. I was drinking every day, and there was not a day that went by without me drinking.

However, over time, many participants said they came to accept their HIV status and learn to cope with the disease. MSM also reported that they had difficulty accepting their sexuality. Some described shame related to having sexual feelings for other men.

Participants reported receiving emotional support from a variety of sources. One MSM said he went to his pastor for support, while another derived comfort from religion but had not disclosed or discussed his life with his church. Only one participant mentioned going to formal counselling services, saying he and his partner saw a private counsellor who knew they were gay. However, most received support from partners, friends or family to whom they had disclosed either their HIV status or their sexual identity.

### Preventing HIV transmission to sexual partners and the context of MSM relationships

MSM in this study were very aware of the need to prevent onward HIV transmission to sexual partners. Many discussed how they had changed their behaviour after being diagnosed with HIV in order to reduce transmission risk to others by using condoms and reducing the number of partners. However, others reported continued risk behaviour, often linked to alcohol use. As one participant put it, “most of the time we have sex without a condom it is when we are drunk.”

Poverty and lack of economic opportunities also shaped risk behaviours. Participants reported that some members of the MSM community were not necessarily gay, but engaged in transactional sex with men to support themselves financially. However, the majority of our participants identified as gay, and many said they were in long-term, monogamous partnerships with other men.

Some MSM felt that the clandestine nature of MSM relationships in Swaziland may lead to greater numbers of and more casual types of partnerships. MSM described many of their partners as bisexual or having female girlfriends and wives, possibly to fulfil cultural expectations. Furthermore, MSM said that their relationships are often kept secret and therefore families do not play a role in relationship counselling and peacekeeping as they might for heterosexual couples.

Usually in our community we have short-term relationships. These relationships are caused by the fact that there is nothing bonding those people. And maybe the community, the parents or relatives are not involved in our relationships. And then if I have got a problem with my boyfriend, if I say it's over, it's over … you are not able to go tell your parents or relatives … if people are informed either way about such people [MSM] in the community, if there is a relationship going on with his parent, the parent will be able to intervene either way, and those relationships will sustain.

### Improving positive health, dignity and prevention services for MSM

MSM said that societal acceptance and stigma reduction would be the most important way to improve services for MSM living with HIV. As one man stated, “If we can be recognized and they can know that there are people who are living this kind of life and they can know how they can reach us in terms of programmes and services.” Participants knew that same-sex relationships were more accepted in neighbouring South Africa and hoped that social norms in Swaziland might shift in a similar direction. They also discussed the organizations working openly for LGBT health and rights in South Africa and noted that the lack of such formal organization in Swaziland limited the ability to develop an effective and appropriate response to HIV for MSM.

Participants held a variety of opinions on how best to tailor existing interventions and services for MSM. Some participants suggested developing special clinics or services for HIV-positive MSM. Others worried that targeted services would reinforce stigma. One potential consideration was including MSM living with HIV as “expert clients” to help navigate HIV treatment services. Participants said less about mental health services; just a handful of interviewees said that increasing access to counsellors would be helpful, as existing HIV care and treatment providers were overworked and did not have time to provide in-depth counselling for PLHIV.

Currently, as there are essentially no HIV-prevention services for MSM in Swaziland, participants suggested a “training of trainers” model, whereby trusted MSM community members could be trained in HIV-prevention messages particularly relevant for MSM and could then share those messages with others in their community. MSM also suggested continued or expanded distribution of condoms and particularly lubricant to prevent condom breakage.

Several participants, both MSM and key informants, said that healthcare workers should be trained on issues related to MSM. As one key informant explained, “Even their procedures manuals should have information on how to handle MARPS [most at-risk populations, including MSM].” Importantly, key informants in this study consistently said that regardless of personal belief, they had an ethical responsibility to provide services to everyone, equally. “As a [member of the] health sector, my belief is non-discriminatory services to all the members of the population, and issues of legality and everything rest with the Ministry of Justice,” said one. Another stated,

Even though I don't approve of what they are doing … as a public health officer, I have to make sure that they have access to health services. I don't have to judge them. I don't have to give my views on what they are doing. But my duty is to make sure that they have access to services … whatever their sexual orientation is, they are human beings, they are Swazi.

## Discussion

This study is among the first studies to examine the positive health, dignity and prevention needs of HIV-positive MSM in sub-Saharan Africa. We found that a social and structural context characterized by significant and multiple stigmas was key to understanding these needs. Dual stigma related to both sexual identity and HIV status led to selective disclosure or lack of disclosure of both identities, and consequently a lack of social support for care-seeking and medication adherence. Perceived and experienced stigma from healthcare settings, particularly around sexual identity, also led to delayed care-seeking, travel to more distant clinics and missed opportunities for appropriate services. These findings support and extend findings from other sub-Saharan African settings that discrimination reduces the willingness of MSM to access services [14–16]. The lack of support from friends, relatives and society for same-sex relationships was described as weakening these relationships, leading to greater numbers of sexual partners as well as relationships with women to “hide” same-sex behaviours, potentially further increasing HIV risk. This finding similarly echoes research from the United States suggesting that psychosocial health problems may increase HIV risk among MSM, leading to a “syndemic” [17]; such findings highlight the need to approach HIV prevention within the context of overlapping health problems [18].

Intersectionality is a theoretical framework that examines the relationship or “intersection” between multiple forms of oppression and discrimination due to social categorizations such as race, class or gender [19]. MSM living with HIV experience the dual stigma of being a sexual minority and having a stigmatizing illness. Intersectionality posits that these multiple stigmas are not experienced independently, but that they interact in complex ways to create disparity and social inequality in health outcomes [19]. We found that MSM living with HIV described dual stigma as an overwhelming burden in their lives which influenced multiple aspects of their health and relationships. Considering the needs of MSM living with HIV in this intersectionality framework provides the deepest understanding of their experience.

Intersectionality also highlights the ways in which individual experiences with stigma reflect larger social structures that create and sustain inequality. Participants in our study experienced outright discrimination, stigma and violence against MSM and PLHIV. However, because sexual identity can be concealed, they often encountered situations in which they were assumed to be heterosexual – assumptions which they did not correct due to fear of discrimination. For example, in healthcare settings, many providers assumed their clients were heterosexual and provided services accordingly. For our participants, these assumptions led to missed opportunities for appropriate counselling services tailored to their individual needs and risks, as well as missed opportunities for offering important services, such as HIV testing and counselling, to their sexual partners. Although the World Health Organization couples HIV testing and counselling guidelines support offering these services to same-sex couples [20], in practice, most couples HIV testing and counselling services in sub-Saharan Africa focus exclusively on steady, heterosexual partnerships and fail to consider same-sex relationships. Although individual providers may offer supportive services for same-sex partners, a more comprehensive approach is needed to incorporate training on same-sex relationships into couples HIV testing and counselling programmes.

Currently, services for MSM living with HIV in Swaziland are essentially non-existent. This is unsurprising, given the lack of data on MSM and HIV risk in Swaziland until very recently and the criminalization of same-sex behaviour in Swaziland. Research has documented a strong correlation between criminalization of same-sex behaviour and lack of investment in services for MSM globally [21]. However, MSM have unique healthcare needs [22], and even in rights-constrained settings, comprehensive HIV services for MSM can and should be provided [23]. Our findings suggest the beginnings of political will among healthcare workers, key stakeholders at the government and local levels and the MSM community to provide these services. Key informants in particular reflected on their duty to provide services to all Swazis in a non-discriminatory manner. These beliefs can provide a foundation for establishing comprehensive HIV services, including both prevention *and* care and treatment services, for MSM. In fact, this research helped, in part, to catalyze the official registration of an NGO, Rock of Hope, dedicated to key population rights in Swaziland, including LGBT rights, which has been invited to engage with the country's key population policy technical working group addressing HIV among MSM and other key populations. The technical working group is under the auspices of the Swaziland National AIDS Programme (SNAP), a programmatic body under the MOH. Other implementing partner organizations providing HIV-related services have indicated they would be open to developing services for MSM. In this changing political and institutional context, there appears to be a genuine possibility of government, NGO and civil society collaboration to develop an effective and comprehensive response to the HIV epidemic among MSM in Swaziland.

This study provides unique information about the needs of MSM living with HIV in a sub-Saharan African context with high HIV disease burden. Conducting multiple interviews with MSM living with HIV and working closely with local LGBT groups increased the comfort level of our participants and their willingness to participate in this study. However, MSM participants were still discussing very sensitive, stigmatized and illegal behaviours, and they may not have fully opened up to interviewers. Data were collected largely from MSM in urban centres due to reliance on existing networks; this may limit transferability of the findings to rural MSM or those without strong MSM social networks.

## Conclusions

The intersecting stigmas of sexual identity and HIV status shaped multiple facets of the lives of MSM living with HIV in Swaziland. Intersectionality provides a framework for understanding these experiences and highlights how programmes and policies should consider the specific needs of this population when designing HIV prevention, care and treatment services. In Swaziland, programmes should consider tailored multi-level interventions that address these unique needs at the policy, societal and healthcare delivery levels. At the policy level, the health sector in Swaziland is already initiating important research to examine the epidemiology and service delivery needs of MSM; findings from this research should be incorporated into the national HIV response. For Swazi society in general as well as healthcare providers, interventions to reduce stigma, discrimination and violence against MSM and PLHIV are needed. The health sector should also consider distributing condoms and lubricant for MSM, training healthcare providers in the specific health needs of MSM and engaging MSM as peer outreach workers or expert clients in both prevention activities and clinical services. Finally, further research examining the experiences and needs of MSM living with HIV globally is required to improve comprehensive HIV services for this population.
